# Back Analysis of Geomechanical Parameters in Underground Engineering Using Artificial Bee Colony

**DOI:** 10.1155/2014/693812

**Published:** 2014-07-17

**Authors:** Changxing Zhu, Hongbo Zhao, Ming Zhao

**Affiliations:** School of Civil Engineering, Henan Polytechnic University, Jiaozuo 454003, China

## Abstract

Accurate geomechanical parameters are critical in tunneling excavation, design, and supporting. In this paper, a displacements back analysis based on artificial bee colony (ABC) algorithm is proposed to identify geomechanical parameters from monitored displacements. ABC was used as global optimal algorithm to search the unknown geomechanical parameters for the problem with analytical solution. To the problem without analytical solution, optimal back analysis is time-consuming, and least square support vector machine (LSSVM) was used to build the relationship between unknown geomechanical parameters and displacement and improve the efficiency of back analysis. The proposed method was applied to a tunnel with analytical solution and a tunnel without analytical solution. The results show the proposed method is feasible.

## 1. Introduction

Numerical analysis plays an important role in construction and design of geotechnical engineering [1]. Geomechanical parameters such as Young's modulus and cohesion are critical to numerical analysis and are difficult to determine because of the complexity and uncertainty of geotechnical engineering. Back analysis is a reliable approach to estimate the geomechanical parameters and is used widely in geotechnical engineering [2]. Because the deformation of rock masses induced by excavation can be measured easily and reliably, displacement-based back analysis techniques as a practical engineering tool are nowadays frequently used in geotechnical engineering problems to determine the unknown geomechanical parameters [3–9].

There are mainly three types of displacement back analysis methods: inverse solving method, atlas method, and direct (i.e., optimal) method [7]. Because of the special advantages, the optimal methods are more and more extensively employed in solving engineering problems [10–12]. Optimization method is important to optimal back analysis. Levenber-Marquardt method, Gauss-Newton method, Bayesian method, Powell method, Rosenbork method, soft computing, and particle swarm optimization have been proposed and applied to back analysis [12–14]. To the practical geotechnical engineering, optimal back analysis needs to call numerical analysis many times. This procedure is time-consuming. Neural network and support vector machine were applied to back analysis to replace the numerical analysis [14–17]. This has been a new way for displacement back analysis.

In this paper, artificial bee colony (ABC) algorithm was chosen for its biological and evolutionary appeal in finding the set of unknown parameters that best matches the modeling prediction with the measured displacement data. Least square support vector machine (LSSVM) was used to replace numerical analysis to present the relationship between unknown geomechanical parameters and displacement of geotechnical structure. Firstly, the idea and algorithm of ABC were presented in Section 2. In Section 3, ABC was adopted to search geomechanical parameters in displacement back analysis. The procedure of ABC-based back analysis was presented to the tunnel with analytical solution and applied to a circular tunnel with hydrostatic stress. Then, to the complex geotechnical engineering without analytical solution, LSSVM was used to present the relationship between geomechanical parameters and displacement. LSSVM model replaced the numerical analysis to improve the efficiency of back analysis. Back analysis based on LSSVM and ABC combination was proposed in Section 4. LSSVM and the procedure of the proposed method were presented in brief. Lastly, some conclusion was listed in Section 5.

## 2. Artificial Bee Colony Algorithms

The artificial bee colony (ABC) algorithm was originally developed in 2005 by Karaboga [18]. In ABC algorithm, the colony of artificial bees contains three groups of bees: employed bees, onlookers, and scouts. Employed bees search for specific food sources (solution) and calculate the amount of nectars (fitness value). Onlooker bees choose a food source based on the nectars shared by employed bees and determine the source to be abandoned and allocate its employed bee as scout bees. Scout bees randomly search for a new food source. The position of a food source represents a possible solution for the problem under consideration and the nectar amount of a food source represents the quality of the solution represented by the fitness value [19, 20]. To the minimum problem, the fitness can be computed by the target function.

In the algorithm, the first half of the colony consists of employed artificial bees and the second half constitutes the onlookers. The number of the employed bees or the onlooker bees is equal to the number of solutions in the population. At the first step, the ABC generates a randomly distributed initial population of* SN* solutions and calculates the fitness of each solution. Consider

(1) $$\begin{array}{c} x\left(i,j\right)=x_{\min}^{j}+\text{rand}\left(0,1\right)\left(x_{\max}^{j}-x_{\min}^{j}\right), \end{array}$$

where *x*(*i*, *j*) is the candidate solution of problem; *i* = 1,2,…, *SN*/2 and *SN*/2 denotes the size of population; *j* = 1,2,…, *D* and *D* is the dimension number of each solution; rand(0,1) is a random number between [0, 1]; *x*_min⁡_^*i*^ and *x*_max⁡_^*i*^ are the upper and lower bound of each solution.

Once initialization is completed, the artificial bees are used to conduct the search for the best food resource (solution). Procedures can be described as follows [20].

- Employed bees determine a food source within the neighborhood of the food source through their memory.
- Employed bees share their information with onlookers within the hive and then the onlookers select one of the food sources.
- Onlookers select a food source within the neighborhood of the food sources chosen by them to produce and exploit the new food resources.
- An employed bee of the sources that have been abandoned by onlookers becomes a scout and starts to search for a new food source randomly.

In the ABC algorithm, a candidate food position can be produced from the memory of bees, which is defined as

(2) $$\begin{array}{c} v\left(i,j\right)=x\left(i,j\right)+\varphi_{ij}\left(x\left(i,j\right)-x\left(k,j\right)\right), \end{array}$$

where* k *used to be different from *i* is randomly chosen indexes from {1, 2, …, *SN*/2},* j* is also randomly chosen indexes from {1,2,…, *D*}, and *φ*_*ij*_ is a random number in [−1,1] and controls the generation of neighbor food sources around *x*(*i*, *j*) and represents the comparison of two food positions seen by a bee. As can be seen from (2), the perturbation on the position *x*(*i*, *j*) decreases when the difference between the parameters of *x*(*i*, *j*) and *x*(*k*, *j*) decreases so that the step length is adaptively reduced.

An artificial onlooker bee chooses a food source based on the probability of food source. The probability of being selected for fitness,* p*_*i*_, can be expressed as

(3) $$\begin{array}{c} p_{i}=\frac{\text{fitnes}{\text{s}}_{i}}{\sum_{n=1}^{SN}\text{fitnes}{\text{s}}_{n}}, \end{array}$$

where fitness_*i*_ is the fitness of the solution.

In ABC algorithm, a food source whose position cannot be improved further through a predetermined number of cycles is assumed to be abandoned by onlookers. *x*(*i*, *j*) used to represent the abandoned source is replaced with *x*′(*i*, *j*) that is a new food source the scout bees find, which is conducted by (1).

Each candidate source position *v*(*i*, *j*) produced by *x*(*i*, *j*) can be evaluated using the comparison between *x*(*i*, *j*) and its old source position. The old food source will be replaced with the new food source when it is equal to or better than the old food source. Otherwise, the old food source is retained in the memory.

There are three control parameters in the ABC, the number of food sources which is equal to the number of employed or onlooker bees (*SN*/2), the value of limit, and the maximum cycle number (MCN). The following is the brief procedure of artificial bee colony (ABC) algorithm.

 
*Step 1.* Determine the value of control parameters* SN*/2, MCN, and “limit” of ABC algorithm.
 
*Step 2.* Generate the initial population *x*(*i*, *j*) by (1) and evaluate the fitness of each solution.
 
*Step 3.* Produce new solution *v*(*i*, *j*) for each employed bee by using (2). In the meantime, the fitness is evaluated.
 
*Step 4.* Calculate the probability *p*_*i*_ for the solution *x*(*i*, *j*) by (3).
 
*Step 5.* Select a solution *x*(*i*, *j*) for each onlooker bee according to *p*_*i*_. Then a new solution *v*(*i*, *j*) is generated by (2).
 
*Step 6.* Calculate the fitness.
 
*Step 7.* If there is an abandoned solution for the scout, it will be replaced by using a new solution which is randomly produced by (2).
 
*Step 8.* Trace the best solution.
 
*Step 9.* Repeat Steps 3 to 8 until the cycle reaches the maximum cycle number (MCN).

## 3. ABC-Based Back Analyses

Optimization algorithm is critical to back analysis. In this section, ABC-based back analysis was presented to identify the geomechanical parameters of a circular tunnel with analytical solution.

### 3.1. The Analytical Solution of Circular Tunnel

**Figure 1:**
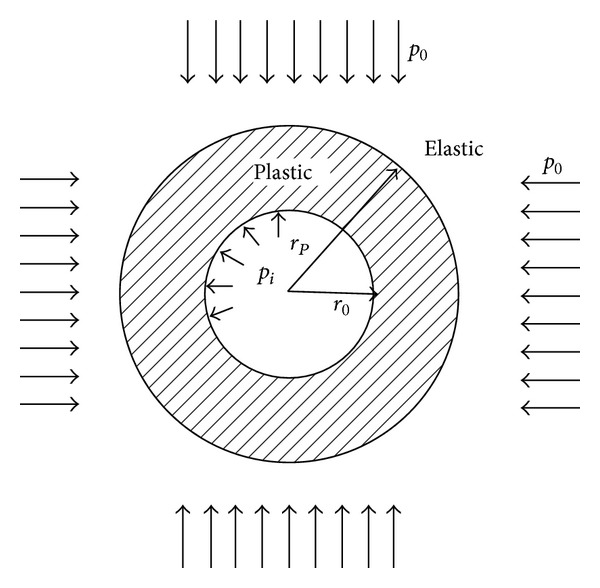
A circular tunnel subjected to hydrostatic far field stress and uniform support pressure.

A circular tunnel is excavated in a continuous, homogeneous, isotropic, initially elastic rock mass and subjected to a hydrostatic far field stress* p*_0_ and uniform support pressure* p*_*i*_ as shown in Figure 1.

According to the Mohr-Coulomb criterion, the normal stress* p*_*cr*_ at the plastic-elastic zone interface is given [21] as follows:

(4) $$\begin{array}{c} p_{cr}=\frac{2p_{o}-\sigma_{c}}{k+1},\\ k=\frac{1+\sin\varphi }{1-\sin\varphi },\\ \sigma_{c}=\frac{c\left(k-1\right)}{\tan\varphi }, \end{array}$$

where  *φ* is the friction angle and* c* is the cohesion. If the uniform support pressure* p*_*i*_ is less than the critical pressure* p*_*cr*_, the plastic zone exists. The plastic zone radius* R* is given [22] as follows:

(5) $$\begin{array}{c} R=r_{o}*{\left[\frac{2\left(p_{o}+s\right)}{\left(k+1\right)\left(p_{i}+s\right)}\right]}^{1/(k-1)} \end{array}$$

in which

(6) $$\begin{array}{c} s=\frac{\sigma_{c}}{k-1} \end{array}$$

and *r*_*o*_ is the radius of the tunnel.

The deformation of surrounding rock of tunnel is as follows.

Elastic zone

(7) $$\begin{array}{c} u_{r}=\frac{\left(p_{o}\sin\varphi +c\cdot \cos\varphi \right)\left(R^{2}/r\right)}{2G}. \end{array}$$

Plastic zone

(8) $$\begin{array}{c} u_{r}=\frac{r}{2G}\cdot \chi , \end{array}$$

where* E* is the deformation modulus and *μ* is Poisson's ratio:

(9) χ=(2μ−1)(po+c·ctgφ) +(1−μ)[(Kp2−1)(Kp+Kps)] ×(pi+c·ctgφ)(Rro)(Kp−1)(Rr)(Kps+1) +[(1−μ)(KpKps+1)(Kp+Kps)·μ] ×(pi+c·ctgφ)(rro)(Kp−1),Kps=(1+sinψs)(1−sinψs),G=E2(1+μ).

### 3.2. Error Function

An error function, in this work, is defined as the minimum error between the displacements predicted by the analytical model based identified parameters and the actual measured displacements. It can be expressed as

(10) $$\begin{array}{c} \text{fitness}=\sqrt{\frac{\sum_{i=1}^{n}{\left(y_{pi}-y_{i}\right)}^{2}}{n}}, \end{array}$$

where* n* is the number of key points, *y*_*i*_ is the monitored displacement of the* i*th key points, and *y*_*pi*_ is the predicted displacement of* i*th key point.

### 3.3. The Procedure of ABC-Based Back Analysis

**Figure 2:**
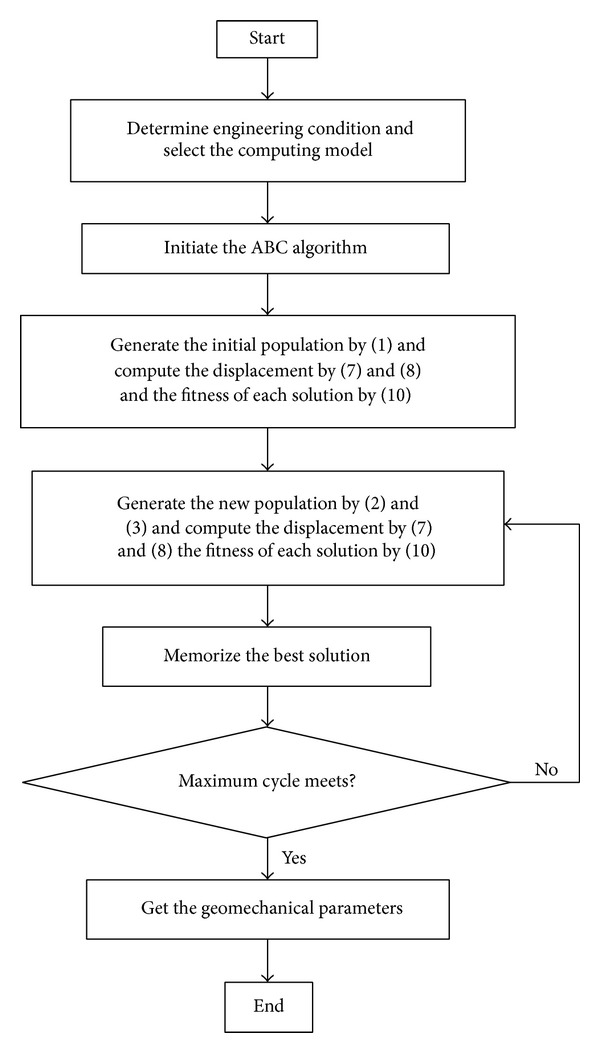
Flowchart of ABC-based back analysis.

ABC-based back analysis is combined ABC with the analytical solution (see (7) and (8)). ABC produces population of artificial bees including employer bees, onlooker bees, and scout bees. The fitness values can be computed by (10). The displacement of (10) can be computed by (7) and (8). Based on the ABC algorithm, the new population was produced. ABC-based back analysis algorithm can be described as follows (see Figure 2).

 
*Step 1.* Collect the information of engineering such as geology conditions and engineering size.
 
*Step 2.* Select the appropriate model according to the above information.
 
*Step 3.* Determine the error function.
 
*Step 4.* Activate the ABC algorithm (see Section 2) to produce the initial population *x*(*i*, *j*) by (1). Displacements are computed using (7) and (8).
 
*Step 5.* The fitness of each solution is calculated by (10).
 
*Step 6*. Generate the new population based on ABC algorithm (see (2) and (3)) and compute the displacement (see (7) and (8)).
 
*Step 7.* Trace the best solution according to the ABC algorithm.
 
*Step 8.* Repeat Steps 5 to 7 until finding the solution or reaching the maximum cycle.

### 3.4. Verification

**Figure 3:**
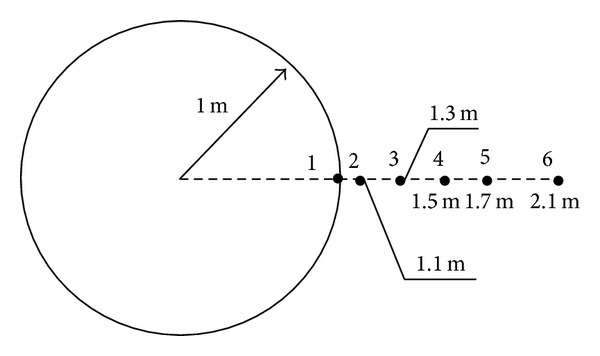
Position of monitored point in circular tunnel.

**Figure 4:**
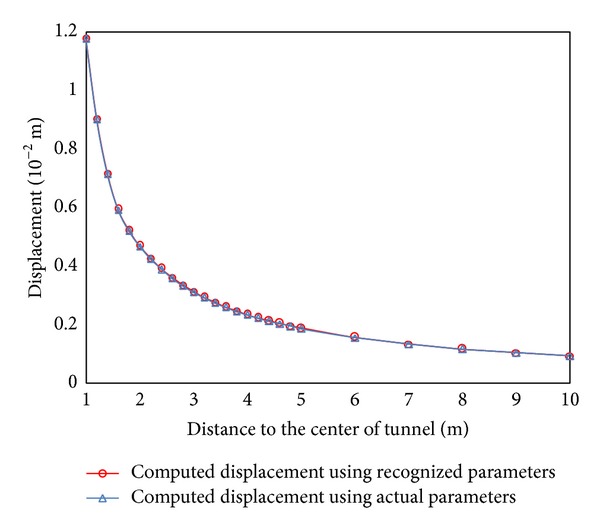
The comparison of displacement between actual and recognized parameters.

**Figure 5:**
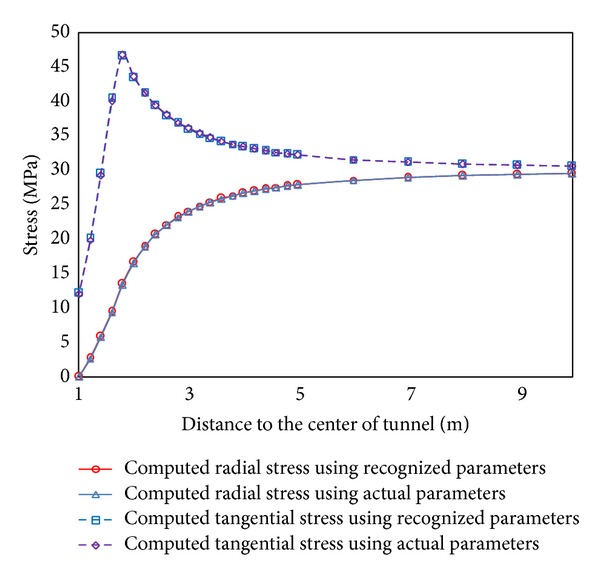
The comparison of stress between actual and recognized parameters.

**Figure 6:**
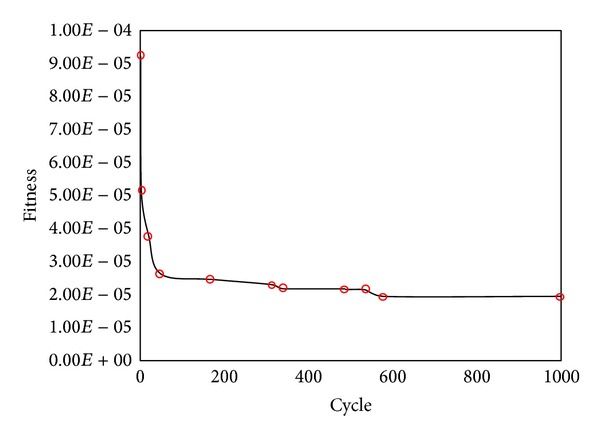
Relationship between fitness value and cycle.

**Figure 7:**
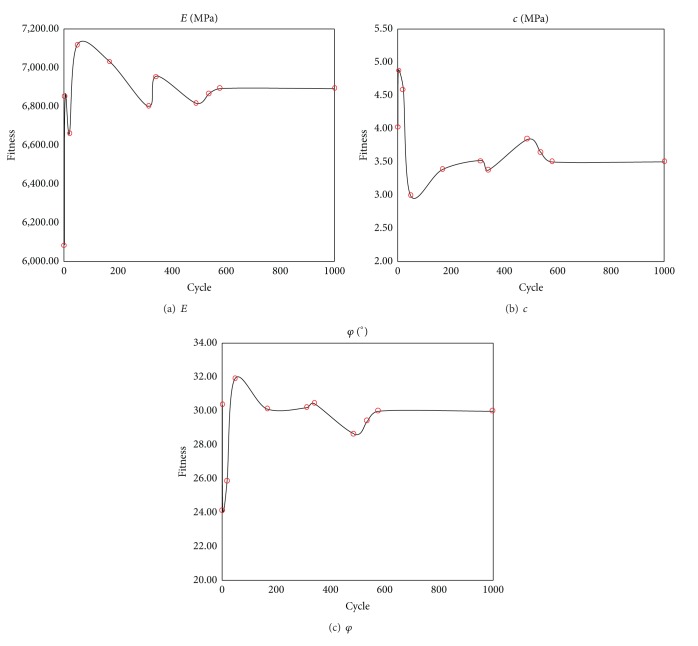
The variation of identified parameter with the cycle.

The displacement of monitored point of tunnel can be computed by the above formula. In this study, six monitored points were used in circular tunnel to monitor the displacements at the horizontal direction for ABC search. The distance between central of tunnel and 6 monitored points is 1.0 m, 1.1 m, 1.3 m, 1.5 m, 1.7 m, and 2.1 m, respectively (see Figure 3). The radius of tunnel is 1 m. The parameter of rock is listed in Table 1. ABC-based back analysis is used to identify geomechanical parameters (e.g., Young's modulus,* E*, cohesion,* c,* and friction angle, *φ*) from displacements of six monitored points. The recognized parameters and their error are listed in Table 2. The maximum relative error is 1.6%. It shows the recognized parameters agree well with the real parameters. The comparison between recognized and real parameters about the displacement and stress of surrounding rock of tunnel is shown in Figures 4 and 5. The results show stresses and displacements of surrounding rock identified by ABC are in well agreement with real stresses and displacements of surrounding rock and ABC is an excellent optimization method. The relationship between fitness and cycle is shown in Figure 6. The relationship between identified parameters and cycle is shown in Figure 7. They show that the performance and convergence of ABC are good and quick for identification of geomechanical parameters using ABC.

#### 3.4.1. Effect of Searching Range

**Figure 8:**
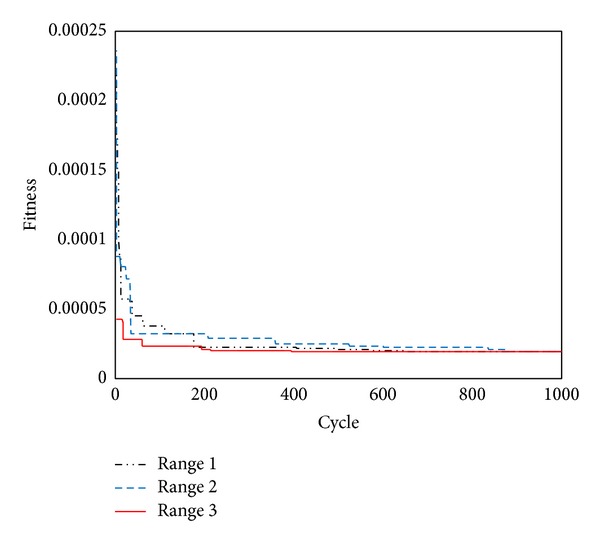
The performance of ABC using different searching ranges.

The performances of ABC are demonstrated with different searching ranges (Table 3). The results of different searching ranges are shown in Figure 8. To the smaller range, the convergence is quicker than the bigger range. But to the bigger range, the fitness is the same as the smaller range. It shows ABC has strong capability of global searching and makes it possible to find the rock mass parameters in a big global space, which enables the back analysis to be applied to more complex engineering problems.

#### 3.4.2. Effect of Population Size

**Figure 9:**
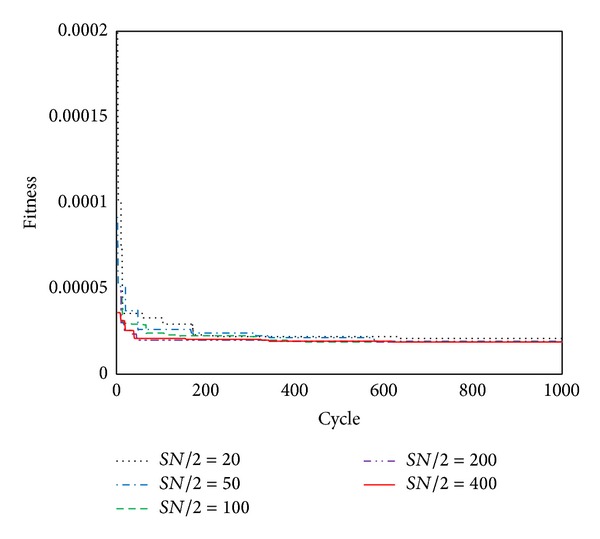
The convergence of different population size.

Population size is key parameters of ABC. To study the effect of the colony size on the convergence rate of the ABC algorithm, five different colonies that consisted of 20, 50, 100, 200, and 400 bees were used. The fitness versus cycle numbers is shown in Figure 9. It can be seen that the convergence rates increase with greater numbers of bees and population size of 200 or 400 bees is enough in this study.

## 4. Back Analysis Based on LSSVM and ABC

In the above section, ABC-based back analysis was used to the circular tunnel with analytical solution. To the practical engineering, it is difficult to get the analytical solution. The procedure with numerical solution is time-consuming. Regression analysis is a good approach to build the relation between geomechanical parameters and field monitored information. In this study, least square support vector machine (LSSVM) was adopted to present the relationship between geomechanical parameters and displacement based on numerical analysis.

### 4.1. Least Square Support Vector Machine

The least square support vector machine (LSSVM) was originally developed by Suykens and Vandewalle [21]. Consider a given training set of* N *data points {*x*_*k*_, *y*_*k*_}  (*k* = 1, 2, …, *N*) with input data* x*_*k*_ *∈* *R* ^*N*^ and output* y*_*k*_ *∈* *r* where* R* ^*N*^ is the* N*-dimensional vector space and* r *is the one-dimensional vector space. According to the LSSVM algorithm, LSSVM model becomes

(11) $$\begin{array}{c} y\left(x\right)=\overset{N}{\underset{k=1}{\sum }}\alpha_{k}K\left(x,x_{k}\right)+b, \end{array}$$

where *K*(*x*, *x*_*k*_) is kernel functions and *α*  and* b *meet the following equation:

(12) $$\begin{array}{c} \left[\begin{array}{cc} 0 & 1^{T}\\ 1 & \Omega +\gamma^{-1}I \end{array}\right]\left[\begin{array}{c} b\\ \alpha \end{array}\right]=\left[\begin{array}{c} 0\\ y \end{array}\right], \end{array}$$

where *y* = [*y*_1_,…, *y*_*N*_], 1 = [1, …, 1], *α* = [*α*_1_, …, *α*_*N*_], and Mercer's theorem is applied within the Ω matrix, Ω = *φ*(*x*_*k*_)^*T*^ *φ*(*x*_*l*_) = *k*(*x*_*k*_, *x*_*l*_), *k*, *l* = 1, …, *N*. Then the analytical solution of *α* and* b *is given by

(13) $$\begin{array}{c} \left[\begin{array}{c} b\\ \alpha \end{array}\right]=\Phi^{-1}\left[\begin{array}{c} 0\\ y \end{array}\right]. \end{array}$$

### 4.2. Representation of Nonlinear Relationship

LSSVM is used in this study to map the nonlinear relationship between geomechanical parameters such as Young's modulus, cohesion, geostress coefficients, and monitored displacements. The mathematical model of least square support vector machine is defined as

(14) $$\begin{array}{c} \text{LSSVM}\left(X\right):R^{n}⟶R,\\ Y=\text{LSSVM}\left(X\right),\\ X=\left(x_{1},x_{2},\ldots ,x_{n}\right),\\ Y=\left(y_{1},y_{2},\ldots ,y_{n}\right), \end{array}$$

where *x*_*i*_(*i* = 1,2,…, *n*) is geomechanical parameters, for example, Young's modulus, friction angle, geostress coefficients, and so forth, and *y*_*i*_(*i* = 1, 2, …, *n*) is displacements of the key points.

In order to obtain LSSVM(**X**), a training process based on the known data set is needed. Necessary training samples are created in this work by using numerical analysis (e.g., FEM model), which is used to obtain displacements of rock mass of key points corresponding to the given set of tentative geomechanical parameters. The geomechanical parameters are defined as input of LSSVM. The displacement is defined as output of LSSVM.

### 4.3. Procedure of Back Analysis Algorithm Based on LSSVM and ABC

After the LSSVM model, representing the nonlinear relation between the displacement and a parameter, is obtained, it can be used to predict displacements at monitored points instead of numerical analysis. ABC is used to search the optimal parameter to be identified based on the error function (see (10)). The back analysis technique based on LSSVM-ABC combination can be described as follows.

 
*Step 1.* Determine ABC parameters and the range of parameters to be recognized.
 
*Step 2.* Generate randomly *n* group of parameters at their given range. Each individual represents an initial solution.
 
*Step 3.* Input a set of rock mass parameters to the model LSSVM(**X**) obtained above to calculate the displacement values at given monitoring points.
 
*Step 4.* Use (10) to evaluate the fitness of the current individuals, that is, the reasonability of the parameter set.
 
*Step 5.* If all individuals are evaluated, then go to Step 6. Otherwise, go to Step 3.
 
*Step 6.* If the maximum cycle is reached or the best individuals (the parameter to be back recognized) are obtained, then the cycle ends and outputs best individuals. Otherwise, go to Step 7.
 
*Step 7.* Update the individuals according to (2) and (3).
 
*Step 8.* Repeat Step 7 until all *n* new individuals are generated. They are used as offspring.
 
*Step 9.* Go to Step 3.

### 4.4. Verification

**Figure 10:**
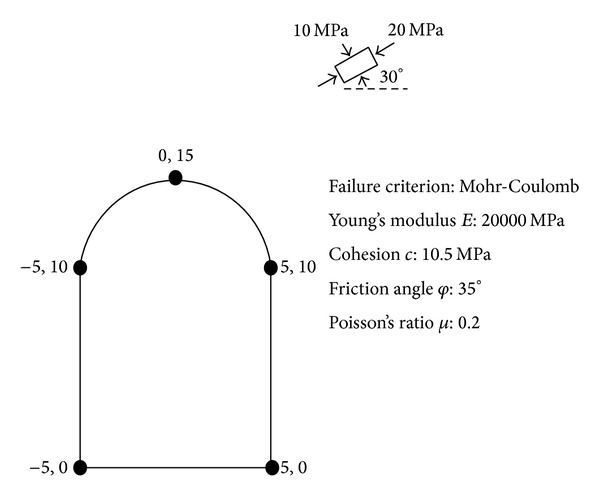
The cross section of tunnel and parameters.

**Figure 11:**
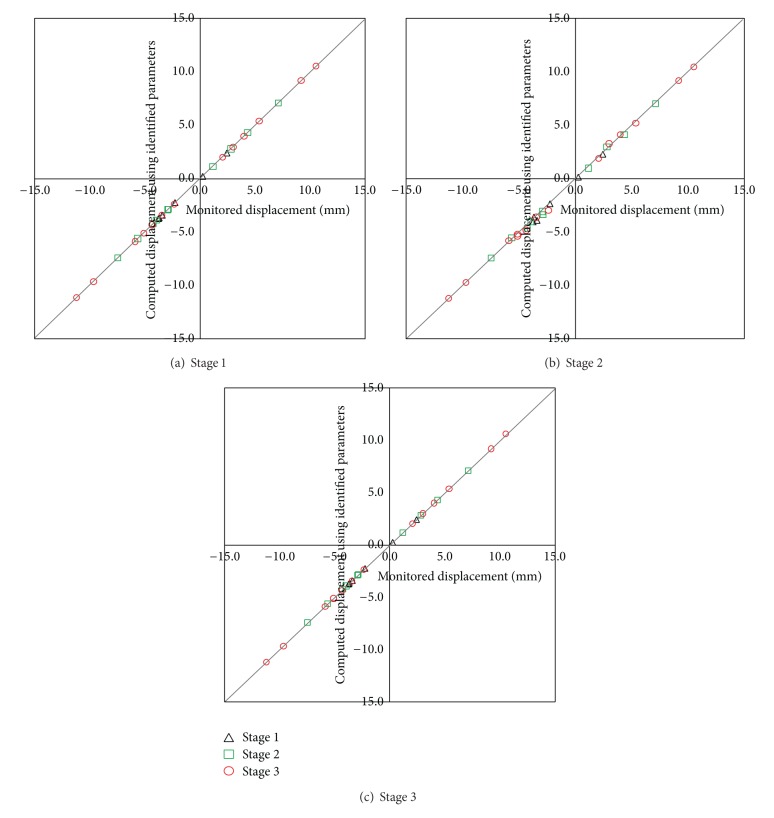
Comparison between monitored displacement and predicted displacement using identified parameters.

**Figure 12:**
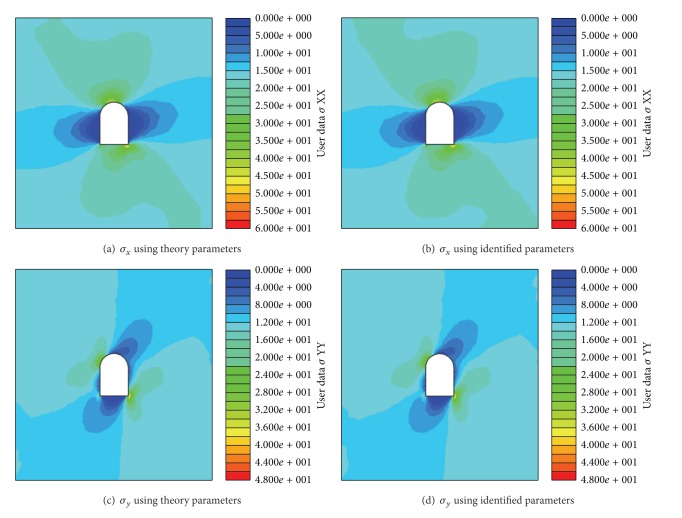
Calculated stress comparison between using theory value and identified value at stage 3.

To verify the model, we suppose there is a tunnel (see Figure 10). The size of tunnel, geomechanical parameters, and in situ stress are listed in Figure 10. The value in Figure 10 is theoretical values. Displacement values for some key points, indicated by nodes, are calculated by elastic finite element method. The suggested algorithm above is used to identify initial geostress components* P*_1_ and* P*_2,_ and angle between* P*_1_ and* P*_2_. We used orthogonal experiment design to create 25 sets of tentative geostresses* P*_1_ and* P*_2_ and angle between* P*_1_ and* P*_2_. The training samples will be obtained through computing the displacement of each set of tentative geostresses. Then the LSSVM model was build based on (13). The training samples and model parameters of LSSVM are listed in Table 5. In situ stresses,* P*_1_ and* P*_2,_ and angle at different stages can be identified according to the procedure of Section 4.3. Identified in situ stress,* P*_1_ and* P*_2, _and angle at different stages are listed in Table 4. The comparison between displacement of the key points using the theoretical parameters and displacements identified by back analysis based on ABC and LSSVM is shown in Figure 11. Stresses of surrounding rock are shown in Figure 12 after stage 3. Results show the proposed method can effectively identify the in situ stress.

### 4.5. Discussions 

#### 4.5.1. Performance of LSSVM

**Figure 13:**
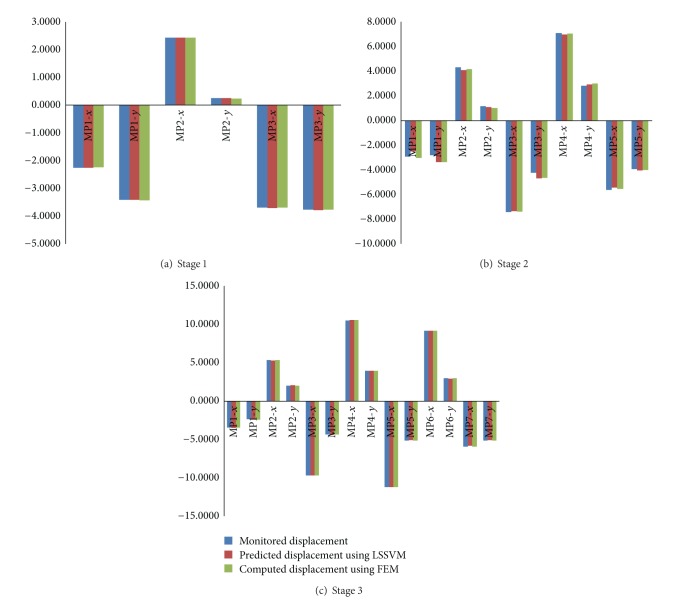
Predicted displacement using LSSVM with calculated displacement using theory and identified parameters.

The performance of LSSVM is very important to back analysis. The predicted displacement using LSSVM is in well agreement with the calculated displacement using theory and identified parameters (shown in Figure 13). It shows the LSSVM model presents well the relationship between geomechanical parameters and displacement. It improves the efficiency of back analysis using LSSVM.

#### 4.5.2. Effect of Kernel Parameters

**Figure 14:**
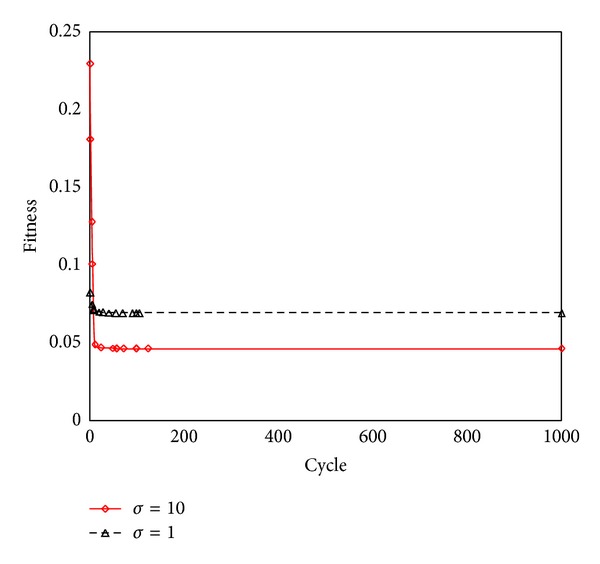
Fitness with different parameters of kernel function.

**Figure 15:**
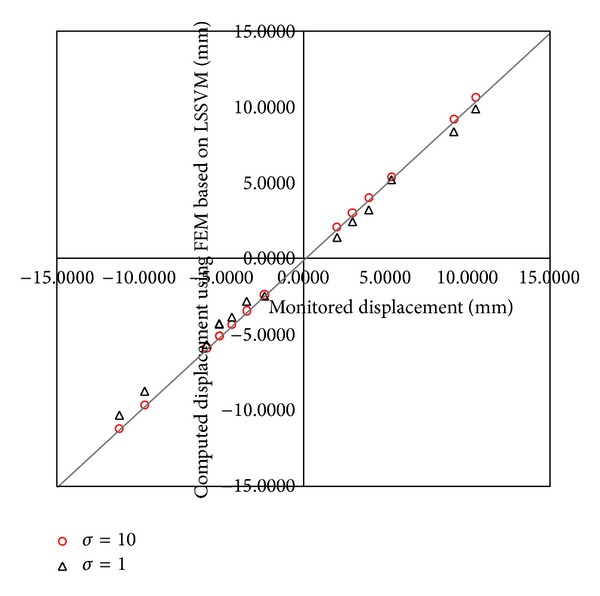
The performance of LSSVM with different parameters of kernel function.

In this study, the RBF kernel function was adopted. The relationship between fitness and cycle is listed in Figure 14 with *σ* = 10 and *σ* = 1. The performance of LSSVM is listed in Figure 15 using *σ* = 10 and *σ* = 1. Its show selecting the appropriate kernel parameters is important to back analysis. But there is not any guide to select kernel function and its parameters according to LSSVM theory. It can be acquired by error-and-trial.

## 5. Conclusions

The paper presents a new methodology called back analysis based on ABC. ABC is used to identify the geomechanical parameters based on monitored displacements. Results of circular tunnel with the analytical solution illustrate clearly that ABC is effectively able to search parameters of geomaterial and has proved ABC has powerful global optimal performance. To improve the efficiency of back analysis, LSSVM was used to present the relationship between geomechanical parameters and displacement instead of numerical analysis. Results of horseshoe tunnel without the analytical solution demonstrate that LSSVM presents well the nonlinear relationship between geomechanical parameters and monitored displacements. The proposed approach improves the efficiency and precision of back analysis and makes it possible to be applied to more complex engineering problem.

## Figures and Tables

**Table 1:** Parameters of tunnel model.

*p*_0_ (MPa)	*E* (MPa)	*c* (MPa)	Φ (°)	*p*_*i*_ (Mpa)	*ψ* (°)
30.0000	7000.0000	3.4500	30.0000	0	0

**Table 2:** Identified parameters using ABC-based back analysis.

	*E* (Mpa)	*c* (Mpa)	*φ* (°)
ABC-based back analysis	6893.04951	3.5065	29.99284
Actual value	7000.0000	3.4500	30.0000
Relative error (%)	1.5279	−1.6377	0.0239

**Table 3:** The ranges of identified parameters.

	Range 1	Range 2	Range 3
E (Mpa)	[2000, 12000]	[4000, 1000]	[5000, 8000]
c (Mpa)	[0.5, 7]	[1, 6]	[3, 7]
*φ* (°)	[5, 60]	[10, 50]	[20, 40]

**Table 4:** Identified in situ stress and angle in different stages.

	*P*_1_	*P*_2_	Angle
Actual	20.0000	10.0000	30.0000
Stage 1	19.9583	10.0614	30.0104
Stage 2	20.6493	10.8171	33.3676
Stage 3	20.0252	10.0376	30.623

**Table 5:** Training samples and model parameters of LSSVM.

Number of samples	*P*_1_ (Mpa)	*P*_2_ (Mpa)	*φ* (°)	Displacement						*α*					
				MP1		MP2		MP3		MP1*x*	MP1*y*	MP2*x*	MP2*y*	MP3*x*	MP3*y*
				*x*	*y*	*x*	*y*	*x*	*y*						
1	10.0000	5.0000	20.0000	−0.8380	−1.3600	1.5500	−0.0231	−2.0200	−1.5100	1.4473	2.0149	−0.8992	−0.3815	1.5989	2.2484
2	10.0000	7.5000	25.0000	−0.4990	−2.3300	1.3900	−0.0687	−1.6700	−1.5800	1.6424	0.8880	−0.9801	−0.3294	1.6348	1.9749
3	10.0000	10.0000	30.0000	0.0000	−3.1300	1.4000	−1.4400	1.4000	−1.4400	2.1479	0.2439	−0.9786	−1.6870	4.9088	2.1843
4	12.5000	12.5000	35.0000	0.0000	−3.9100	1.7500	−1.8000	−1.7500	−1.8000	2.0307	−0.3980	−0.5684	−1.8560	1.4959	1.7655
5	15.0000	15.0000	40.0000	−0.0001	−4.7000	2.0900	−2.1600	−2.1000	−2.1700	2.0040	−1.0849	−0.2202	−2.1514	1.2404	1.4127
6	15.0000	5.0000	25.0000	−2.0000	−1.4700	2.0800	0.8610	−3.1900	−2.7200	0.2187	1.8194	−0.3108	0.5215	0.3286	0.9391
7	15.0000	7.5000	30.0000	−1.6800	−2.5600	1.8300	0.1890	−2.7700	−2.8200	0.5089	0.6915	−0.5137	−0.1191	0.5530	0.7972
8	15.0000	10.0000	35.0000	−1.2300	−3.4700	1.7400	−0.5740	−2.4200	−2.7500	0.6722	0.0683	−0.5353	−0.5142	0.7871	0.7058
9	15.0000	12.5000	40.0000	−0.6420	−4.1900	1.8300	−1.3800	−2.1800	−2.5200	1.0483	−0.3389	−0.5100	−1.0033	1.0326	0.8752
10	15.0000	15.0000	20.0000	−0.0001	−4.7000	2.0900	−2.1600	−2.1000	−2.1700	2.2964	−1.2063	−0.4593	−2.4334	1.6207	1.6580
11	20.0000	5.0000	30.0000	−3.4100	−1.9500	2.2700	1.8500	−4.2500	−4.3300	−0.9584	1.4147	−0.1741	1.3821	−0.5279	−0.4169
12	20.0000	7.5000	35.0000	−3.0700	−3.2100	1.9200	1.1000	−3.6000	−4.3700	−0.4940	0.2093	−0.4605	0.5409	0.1538	−0.3109
13	20.0000	10.0000	40.0000	−2.5800	−4.2600	1.7400	0.2750	−3.1500	−4.3100	−0.1430	−0.6938	−0.6499	−0.1060	0.5071	−0.3365
14	20.0000	12.5000	20.0000	−1.2600	−3.6100	3.0300	−0.7560	−3.7300	−2.9900	0.9442	−0.0545	0.4200	−0.9120	0.0125	0.7845
15	20.0000	15.0000	25.0000	−0.9990	−4.6500	2.7900	−1.3700	−3.3400	−3.1500	1.2917	−1.1019	0.1791	−1.6037	0.4438	0.6994
16	25.0000	5.0000	35.0000	−5.0300	−2.8100	2.2000	2.9600	−5.3200	−6.2900	−2.3159	0.7126	−0.2344	2.3232	−1.4578	−2.0741
17	25.0000	7.5000	40.0000	−4.5700	−4.3400	1.7200	2.0100	−4.2700	−6.2200	−1.7211	−0.8042	−0.7054	1.2559	−0.3011	−1.8612
18	25.0000	10.0000	20.0000	−2.5600	−2.5500	4.0000	0.6760	−5.3900	−3.8400	−0.0681	0.8422	1.2115	0.2427	−1.3147	0.1347
19	25.0000	12.5000	25.0000	−2.5800	−3.8300	3.5200	0.2050	−4.8900	−4.2900	−0.1085	−0.2739	0.7151	−0.1537	−0.8165	−0.2612
20	25.0000	15.0000	30.0000	−2.3100	−5.0100	3.2000	−0.3910	−4.4100	−4.5200	0.2387	−1.3780	0.5252	−0.8035	−0.4328	−0.4061
21	30.0000	5.0000	40.0000	−7.0100	−4.2700	1.9200	4.2500	−6.3900	−8.5200	−4.4142	−0.7485	−0.5555	3.7206	−2.5680	−4.4067
22	30.0000	7.5000	20.0000	−4.1800	−1.5000	5.1000	2.1700	−7.4200	−4.8300	−1.6564	1.9159	2.3915	1.6943	−3.4243	−0.8180
23	30.0000	10.0000	25.0000	−4.3200	−3.0600	4.3800	1.8900	−6.5200	−5.5400	−1.6996	0.3741	1.5582	1.3646	−2.3453	−1.4311
24	30.0000	12.5000	30.0000	−4.1800	−4.5200	3.7600	1.3100	−5.8600	−5.9500	−1.5641	−0.8404	1.0366	0.8632	−1.8027	−1.7517
25	30.0000	15.0000	35.0000	−3.8900	−5.8500	3.3200	0.5890	−5.2300	−6.1900	−1.3480	−2.2716	0.7182	0.1455	−1.3269	−2.1053
*b*	—	—	—	—	—	—	—	—	—	−2.4124	−3.4816	2.5241	0.3809	−3.7541	−3.9253
